# Differences between resident self-assessments and faculty- assessments on Anesthesiology Milestones and associated factors: a cross-sectional study

**DOI:** 10.1186/s12909-024-05544-6

**Published:** 2024-05-17

**Authors:** Xiaohan Xu, Xia Ruan, Chunhua Yu, Xuerong Yu, Xiang Quan, Xu Li, Tangmi Yuan, Di Xia, Yuelun Zhang, Lijian Pei

**Affiliations:** 1 Department of Anesthesiology, Peking Union Medical College Hospital, Chinese Academy of Medical Science, Peking Union Medical College, Beijing, 100730 China; 2 Centre for Prevention and Early Intervention, National Infrastructures for Translational Medicine, Institute of Clinical Medicine, Peking Union Medical College Hospital, Chinese Academy of Medical Science, Peking Union Medical College, Beijing, 100730 China

**Keywords:** Anesthesiology, Residency training, Self-assessment, Clinical Competency Committee, ACGME Milestone

## Abstract

**Background:**

Accurate self-assessment is crucial for the professional development of physicians. There has been sparse data on the accuracy of self-assessments on Anesthesiology Milestones. The aim of this study was to investigate the differences between resident self-assessments and faculty-assessments on Anesthesiology Milestones and the associated factors.

**Methods:**

This was a cross-sectional study conducted in a general tertiary university-affiliated hospital. We included anesthesia residents who were enrolled in the standardized residency training program in postgraduate year two and three at the time of the Milestone evaluation. We requested evaluations of competencies from both the Clinical Competency Committee faculty and the anesthesia residents themselves, utilizing the Chinese version of Anesthesiology Milestones in January 2023 and January 2024. The primary outcome was the differences between self- and faculty-assessments, calculated by subtracting the faculty-rated score from the self-rated score on each subcompetency.

**Results:**

A total of 46 and 42 residents were evaluated in year 2023 and 2024, respectively. The self-rated sum score was significantly higher than that rated by faculty [mean (standardized deviation): 120.39 (32.41) vs. 114.44 (23.71), *P* = 0.008 in paired t test] with an intraclass correlation coefficient of 0.55 [95% confidence interval (CI): 0.31 to 0.70]. The Bland–Altman plots revealed significant overestimation in patient care (bias 0.32, 95% CI: 0.05 to 0.60), practice-based learning and improvement (bias 0.45, 95% CI: 0.07 to 0.84), and professionalism (bias 0.37, 95% CI: 0.02 to 0.72). Ratings from residents with master’s degrees (mean difference: -1.06, 95% CI: -1.80 to -0.32, *P* = 0.005) and doctorate degrees (mean difference: -1.14, 95% CI: -1.91 to -0.38, *P* = 0.003) were closer to the faculty-assessments than residents with bachelor's degrees. Compared with patient care, the differences between self- and faculty- rated scores were smaller in medical knowledge (mean difference: -0.18, 95% CI: -0.35 to -0.02, *P* = 0.031) and interpersonal and communication skills (mean difference: -0.41, 95% CI: -0.64 to -0.19, *P* < 0.001) in the generalized estimating equation logistic regression model.

**Conclusions:**

This study revealed that residents tended to overestimate themselves, emphasizing the need to improve the accuracy of Milestones self-assessment. The differences between self- and faculty-assessments were associated with residents’ degrees and domains of competency.

## Background

Accurate self-assessment is crucial for the professional development of physicians [1, 2]. It helps them identify their own strength and weakness, set realistic expectations, and continue self-directed lifelong learning [3]. In spite of these benefits, previous studies on various medical specialties have suggested the inconsistency between self-assessments and external measures, such as expert assessments or objective examinations [2, 4–9]. Therefore, it is necessary to discover factors that may affect the accuracy of self-assessment and develop strategies to ameliorate this inconsistency.

The Anesthesiology Milestone 2.0 has been developed by the Accreditation Council for Graduate Medical Education (ACGME) to assess competency acquisition of anesthesia residents in the United States (US), which provided descriptions of behaviors that residents are expected to demonstrate as they progress through training in six domains of competencies, including patient care (PC), medical knowledge (MK), system-based practice (SBP), practice-based learning and improvement (PBLI), professionalism (PROF), and interpersonal and communication skills (ICS) [10]. Each residency training programs is required to establish a Clinical Competency Committee (CCC), which is responsible to rate each resident using the Anesthesiology Milestones every six months in the US [11]. In recent years, many residency training programs have encouraged residents to assess themselves using the ACGME Milestones [9, 12–17]. Realizing the differences between self- and CCC-assessments can effectively reinforce the understanding of the Milestone Evaluation System and improve the ability of reflective practice. However, there has been sparse data on the comparison between self- and CCC-assessments on Anesthesiology Milestones.

The aim of this study was to evaluate the differences between resident self- and faculty- assessments on Anesthesiology Milestones and to investigate the associated factors.

## Methods

This was a single-center cross-sectional study conducted at Peking Union Medical College Hospital (PUMCH), a general tertiary university-affiliated teaching hospital in Beijing, China. The institutional review board of PUMCH classified our study as "exempt" and waived the requirement of written informed consent. This article adheres to Strengthening the Reporting of Observational Studies in Epidemiology (STROBE) guideline.

### Study population

We included anesthesia residents who were enrolled in the standardized residency training program in postgraduate year (PGY) two and three at the time of the Milestone evaluation. The medical education programs vary widely in China. After high school graduation, there are two major pathways to pursue a clinical medical degree: the 5-year program leading to a Bachelor of Medicine degree and the 8-year program leading to a Doctor of Medicine degree [18]. The 8-year programs are more favored for offering extensive clinical rotations, research opportunities, and enhanced employment prospects; consequently, they require higher admission scores in the National College Entrance Examination. Upon passing the National Graduate School Entrance Examination, graduates from 5-year medical programs have the option to enroll in a Master of Medicine program. During this period, they undergo residency training and engage in research. Graduates from 5-year programs who did not pass the examination, as well as graduates from 8-year programs, are also required to complete standardized residency training before the promotion to attending physicians. The anesthesiology residency training program in China spans three years, consisting of a 9-month rotation in anesthesia related departments including medical, surgical, and intensive care departments, followed by 27-month rotation in various subspecialties of anesthesiology and pain medicine [19]. Residents in PGY1 spent the majority of their first year rotating through anesthesia related departments and were consequently excluded from this evaluation.

### Development and validation of the Chinese version of Anesthesiology Milestone

The Anesthesiology Milestone 2.0 [20] was translated into Chinese by two professors of anesthesiology and a professor of English literature. There were a total of 23 subcompetencies and five milestone levels in each subcompetency. Tracking from level 1 to level 5 was synonymous with moving from novice to expert resident in the subcompetency. A numeric rating scale of 0–9 were used (0: not yet assessable, 1: not yet completed level 1, 2: equal to level 1, 3: between level 1 and 2, 4: equal to level 2…… 10: equal to level 5). The CCC of our program consisted of 8 anesthesiologists. Five of them had more than 15 years of experience in postgraduate education, while the remaining three possessed 7–8 years of experience. They specialized in diverse subspecialties such as cardiac, thoracic, obstetric, pediatric, and regional anesthesia, enabling them to assess residents comprehensively across various subcompetencies. The CCC faculty assessed 64 anesthesia residents using the Chinese version of Anesthesiology Milestone 2.0 in the year 2022. Their ratings demonstrated satisfactory inter-rater reliability, internal consistency, and correlation with written examination scores.

### Data collection

We requested the CCC faculty to evaluate anesthesia residents in PGY2 and PGY3 on the 23 subcompetencies using the Chinese version of Anesthesiology Milestones 2.0 in January 2023 and January 2024. All the CCC faculty members supervised residents in PGY2 and PGY3 in daily work and were thus familiar with their performances. Before assessing the residents, they underwent training in the use of Anesthesiology Milestones 2.0 based on the Supplemental Guide issued by ACGME [21]. They collaboratively discussed the ratings for each resident until a consensus was reached. During these discussions, scores from daily supervisor evaluations, quarterly written examinations and annal Objective Structured Clinical Examinations were provided. At the same time, the residents in PGY2 and PGY3 were also requested to select the level that best described their own performance on each subcompetency using the same version of Milestones. The faculty and residents were blinded to each other’s rating scores during this process.

We also collected data of variables that may be associated with the accuracy of Milestone assessments, including age, gender, grade, evaluation year, medical education degree, and rank of written examination scores. The written examinations were conducted every three months, composed of case-based single- and multiple-choice questions. In this study, we initially standardized the scores of each written examination using z scores (subtracting each resident’s score from the average score of all the residents in an examination and dividing this by the standard deviation). Subsequently, we ranked the residents based on their mean standardized score of all the written examinations within one year before the evaluation.

### Outcome measure

The primary outcome was the differences between self- and faculty-assessments, measured by subtracting CCC-rated scores from self-rated scores on each subcompetency of Anesthesiology Milestone 2.0.

### Statistical analysis

Normally distributed continuous variables and categorial variables were described as mean (standardized deviation) and number (percentage), respectively. The differences between self- and faculty-rated scores on each subcompetency were analyzed using paired Student’s t test, as suggested by the normal distribution indicated by histograms. The consistency between them was analyzed using intraclass correlation coefficient (ICC) which was estimated by two-way mixed-effects models on absolute agreements. The Bland–Altman plots were used to assess the agreement between the self- and faculty-rated scores within each competency.

The assessment of each subcompetency was considered as an observation in the analysis of factors associated with the differences between self- and faculty-assessments. The association was analyzed using a multivariable generalized estimating equation (GEE) linear model with a robust standard error estimate to account for the clustering effects of assessments on different subcompetencies in the same resident. In addition to the variables related to resident characteristics, the domain of competency was also included into the multivariable model as an independent variable, since residents might rate themselves higher or lower in certain domains. Independent, autoregressive 1, and exchangeable working correlation structures were all used. The independent working correlation structure was finally selected since it had the smallest quasi-likelihood information criterion indicating the best fitness.

In the power analysis, the design effect (D) was calculated by D = 1 + ICC (m-1) [22]. The ICC was estimated using a linear mixed effects model, in which the faculty’s rating on each subcompetency was the dependent variable and the resident was the random intercept. In our study, m was the number of subcompetencies per resident. An ICC of 0.53 and an m of 23 resulted in a design effect of 12.6. The sample size (N) was then calculated by N = m × number of residents/ D. Since there were 88 residents evaluated in both years, the sample size was 160. Based on a two-sided probability of the type I error of 0.05, the statistical power was 82.8% to detect a mean difference between self- and faculty-rated Milestone scores of 0.3 with a pooled standardized deviation of 1.3.

A two-sided *P* < 0.05 was considered statistically significant. The statistical analysis was carried out using R (version 4.2.1, R Foundation for Statistical Computing, Vienna, Austria, 2022) with the packages of irr, blandr, geepack, lme4, and pwr.

## Results

A total of 46 and 42 residents were enrolled in the residency training program in the PGY2 and PGY3 grades at PUMCH in January 2023 and January 2024, respectively. Data of the faculty- and self-assessments on Anesthesiology Milestone 2.0 were collected and analyzed from all these residents. Notably, only 64 distinct residents participated in the evaluation, as 24 residents were in PGY2 during the first evaluation and transitioned to PGY3 for the second evaluation, resulting in their repeated assessments. Table 1 provides further details of the residents’ characteristics.

**Table 1:** Characteristics of residents

Characteristics	Evaluation in 2023 (*N* = 46)	Evaluation in 2024 (*N* = 42)
Age (year)	27.1 (2.4)	27.3 (2.9)
Gender		
Male	14 (30.4%)	14 (33.3%)
Female	32 (69.6%)	28 (66.7%)
Grade		
PGY2	24 (52.2%)	18 (42.9%)
PGY3	22 (47.8%)	24 (57.1%)
Education		
Bachelor	19 (41.3%)	8 (19.0%)
Master	18 (39.1%)	25 (59.5%)
Doctor	9 (19.6%)	9 (21.4%)
Standardized score of written examination	0 (0.67)	0 (0.76)

*Abbreviation*: *PGY* postgraduate year

^a^Continuous variables were described as mean (standardized deviation), and categorial variables were described as number (percentage)

**Fig. 1:**
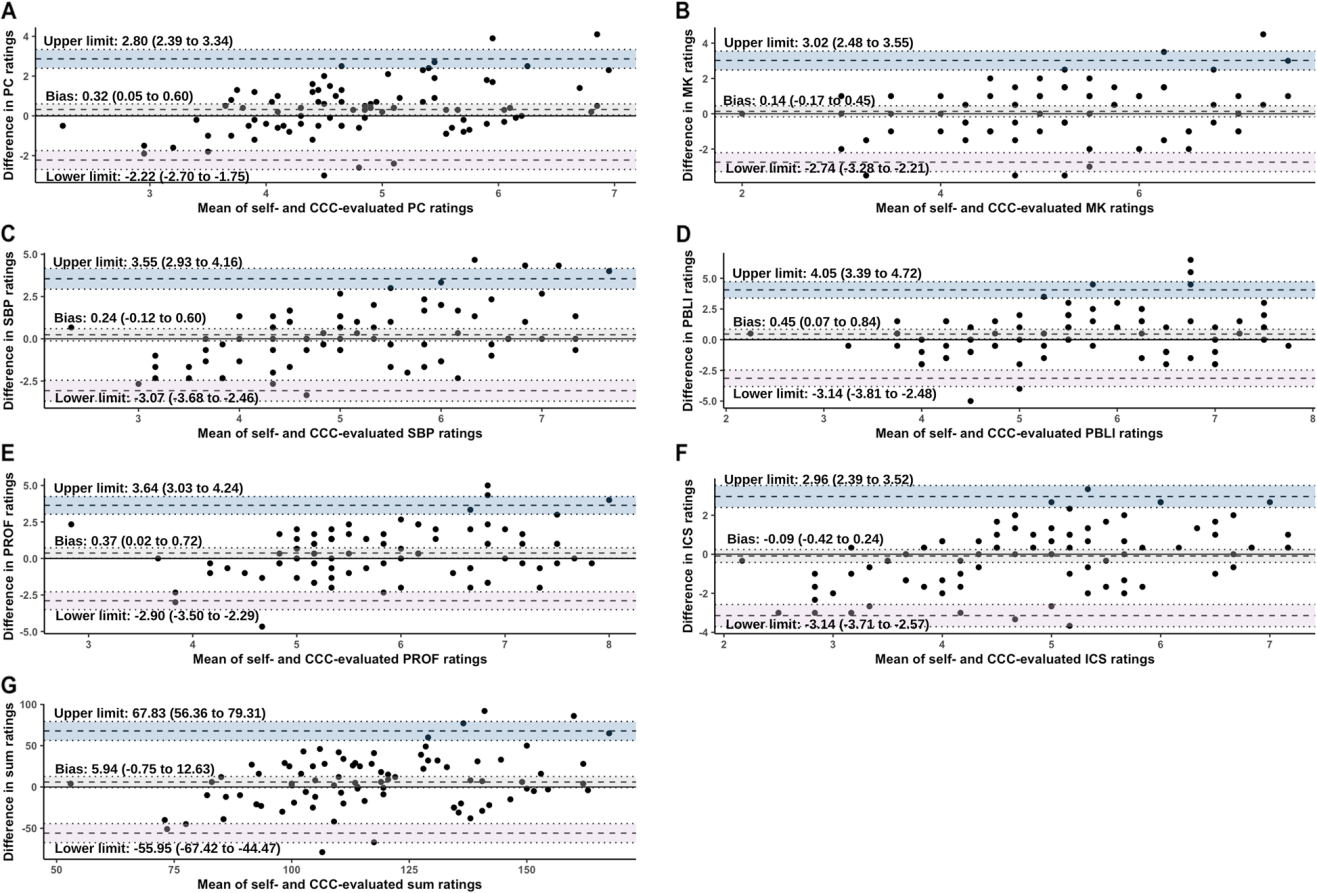
Bland–Altman plots of self- and Clinical Competency Committee- evaluated Milestone rating scores. **A** patient care, **B** medical knowledge; **C** systems-based practice; **D** practice-based learning and improvement; **E** professionalism; **F** interpersonal and communication skills; **G** sum score. Grey area: bias and 95% confidence intervals, blue area: upper limit of agreement and 95% confidence intervals; pink area: lower limit of agreement and 95% confidence intervals

Table 2 summarizes self- and faculty- rated scores on the 23 subcompetencies of Anesthesiology Milestone 2.0. The self-rated sum score was significantly higher than that rated by faculty [mean (standardized deviation, SD): 120.39 (32.41) vs. 114.44 (23.71), *P* = 0.008 in the paired t test] with an ICC of 0.55 [95% confidence interval (CI): 0.31 to 0.70] that did not indicate a strong consistency. Residents’ ratings were significantly higher than faculty’s ratings on 10 subcompetencies and significantly lower on foundational knowledge. The ICCs varied widely from 0.22 (95% CI: -0.17 to 0.49) of PROF3 to 0.60 (95% CI: 0.40 to 0.74) of ICS2 among all the subcompetencies. The Bland–Altman plots (Fig. 1) showed that residents rated significantly higher than faculty in PC (bias 0.32, 95% CI: 0.05 to 0.60), PBLI (bias 0.45, 95% CI: 0.07 to 0.84), and PROF (bias 0.37, 95% CI: 0.02 to 0.72). The bias of the sum rating was 5.94 (95% CI: -0.75 to 12.63) with a lower limit of agreement of -55.95 (95% CI: -67.42 to -44.47), an upper limit of agreement of 67.83 (95% CI: 56.36 to 79.31) and 5 (5.7%) outliers beyond both limits.

**Table 2:** Comparison between self- and faculty-assessments on Anesthesiology Milestones (*N* = 88)

Subcompetency	Self-assessments^a^	Faculty-assessments^a^	t test^b^	ICC	
			*P*	Estimate	95% CI
PC1: Pre-Anesthetic Evaluation	5.77 (1.53)	5.60 (1.09)	0.285	0.54	0.30 to 0.70
PC2: Peri-Operative Care and Management	4.98 (1.59)	4.68 (0.89)	0.081	0.41	0.10 to 0.61
PC3: Application and Interpretation of Monitors	5.40 (1.51)	4.83 (1.00)	< 0.001^*^	0.52	0.23 to 0.69
PC4: Intra-Operative Care	5.61 (1.44)	5.31 (1.11)	0.055	0.50	0.24 to 0.67
PC5: Airway Management	5.35 (1.63)	4.85 (0.88)	0.003^*^	0.46	0.18 to 0.65
PC6: Point-of-Care Ultrasound	3.74 (1.69)	2.90 (0.83)	< 0.001^*^	0.32	-0.06 to 0.56
PC7: Situational Awareness and Crisis Management	4.69 (1.53)	4.73 (1.05)	0.840	0.43	0.13 to 0.63
PC8: Post-Operative Care	5.05 (1.68)	4.94 (1.00)	0.551	0.49	0.23 to 0.67
PC9: Critical Care	4.94 (1.70)	4.56 (1.14)	0.031^*^	0.51	0.25 to 0.68
PC10: Regional Anesthesia	4.44 (1.69)	4.38 (0.97)	0.721	0.28	-0.11 to 0.53
MK1: Foundational Knowledge	5.07 (1.51)	5.47 (1.07)	0.009^*^	0.60	0.38 to 0.74
MK2: Clinical Reasoning	5.59 (1.96)	4.92 (1.42)	0.003^*^	0.42	0.12 to 0.62
SBP1: Patient Safety and Quality Improvement	5.66 (1.77)	5.03 (1.23)	0.003^*^	0.34	0.01 to 0.57
SBP2: System Navigation for Patient-Centered Care	4.70 (1.94)	4.50 (1.05)	0.339	0.31	-0.06 to 0.55
SBP3: Physician Role in Health Care Systems	5.52 (2.04)	5.64 (1.21)	0.584	0.50	0.24 to 0.67
PBLI1: Evidence-Based and Informed Practice	5.69 (2.04)	5.24 (1.49)	0.048^*^	0.45	0.16 to 0.64
PBLI2: Reflective Practice and Commitment to Personal Growth	5.83 (1.60)	5.38 (1.27)	0.024^*^	0.27	-0.01 to 0.52
PROF1: Professional Behavior and Ethical Principles	5.67 (1.72)	5.82 (1.10)	0.443	0.37	0.03 to 0.59
PROF2: Accountability/ Conscientiousness	6.48 (1.74)	5.67 (1.29)	< 0.001^*^	0.33	-0.02 to 0.56
PROF3: Well-Being	5.85 (1.73)	5.40 (1.21)	0.033^*^	0.22	-0.17 to 0.49
ICS1: Patient- and Family-Centered Communication	5.03 (1.89)	5.09 (1.06)	0.766	0.49	0.22 to 0.67
ICS2: Interprofessional and Team Communication	4.94 (1.79)	5.12 (1.16)	0.292	0.60	0.40 to 0.74
ICS3: Communication within Health Care Systems	4.36 (1.81)	4.40 (1.22)	0.857	0.52	0.26 to 0.69
Sum	120.39 (32.41)	114.44 (23.71)	0.008^*^	0.55	0.31 to 0.70

*Abbreviations*: *ICC* interclass correlation coefficient, *CI* confidence interval, *PC* patient care, *MK* medical knowledge, *SBP* systems-based practice, *PBLI* practice-based learning and improvement, *PROF* professionalism, *ICS* interpersonal and communication skills

^*^P < 0.05

^a^Described as mean (standard deviation)

^b^analyzed by paired t test

Table 3 demonstrates the results of GEE logistic regression models of the differences between self- and faculty-assessments. Medical education degree and domain of competency were significantly associated with the differences between self- and faculty- rated Milestone scores in the multivariable model (Table 3). Ratings from residents with master’s degrees (mean difference: -1.06, 95% CI: -1.80 to -0.32, *P* = 0.005) and doctorate degrees (mean difference: -1.14, 95% CI: -1.91 to -0.38, *P* = 0.003) were closer to the faculty-assessments, when compared to residents with bachelor's degrees. Compared with the PC competency, the differences between self- and faculty- rated scores were smaller in MK (mean difference: -0.18, 95% CI: -0.35 to -0.02, *P* = 0.031) and ICS (mean difference: -0.41, 95% CI: -0.64 to -0.19, *P* < 0.001). The multivariable model did not detect any significant associations between age, gender, grade, or ranks of written examination scores and the differences between self- and faculty-assessments.

**Table 3:** Generalized estimating equation logistic regression models of the differences between self- and faculty-assessments on Anesthesiology Milestones (*N* = 2024)

Variable	Difference between self- and faculty-assessments^a^	Univariable Model			Multivariable Model		
		Estimated mean difference	95% CI	*P*	Estimated mean difference	95% CI	*P*
Age (year)	0.62 (1.88)	-0.22	-0.48 to 0.04	0.095	-0.06	-0.20 to 0.08	0.412
Gender							
Female	0.21 (1.67)	Reference			Reference		
Male	0.36 (1.95)	0.15	-0.52 to 0.83	0.655	0.42	-0.25 to 1.09	0.216
Year							
2023		Reference			Reference		
2024		-0.31	-0.82 to 0.20	0.239	0	-0.50 to 0.50	0.995
Grade							
PGY2	0.36 (1.71)	Reference			Reference		
PGY3	1.17 (1.81)	-0.19	-0.69 to 0.32	0.469	-0.24	-0.79 to 0.30	0.381
Degree							
Bachelor	0.95 (1.54)	Reference			Reference		
Master	-0.08 (1.84)	-1.03	-1.64 to -0.42	0.001^*^	-1.06	-1.80 to -0.32	0.005^*^
Doctor	-0.04 (1.60)	-0.90	-1.60 to -0.21	0.011^*^	-1.14	-1.91 to -0.38	0.003^*^
Competency							
PC	0.32 (1.59)	Reference			Reference		
MK	0.14 (1.82)	-0.18	-0.35 to -0.02	0.031^*^	-0.18	-0.35 to -0.02	0.031^*^
SBP	0.24 (1.96)	-0.08	-0.27 to 0.11	0.403	-0.08	-0.27 to 0.11	0.403
PBLI	0.46 (1.99)	0.13	-0.13 to 0.40	0.317	0.13	-0.13 to 0.40	0.317
PROF	0.37 (1.93)	0.05	-0.18 to 0.29	0.673	0.05	-0.18 to 0.29	0.673
ICS	-0.09 (1.72)	-0.41	-0.64 to -0.19	< 0.001^*^	-0.41	-0.64 to -0.19	< 0.001^*^
Rank of Standardized Scores of Written Examinations							
0–25%	1.22 (1.79)	Reference			Reference		
25–50%	0.16 (1.34)	-1.07	-1.75 to -0.38	0.002^*^	-0.08	-0.85 to 0.70	0.844
50–75%	0.30 (1.74)	-0.92	-1.71 to -0.14	0.021^*^	0.48	-0.32 to 1.28	0.240
75–100%	-0.65 (1.65)	-1.87	-2.70 to -1.04	< 0.001^*^	-0.67	-1.99 to 0.65	0.317

*Abbreviations*: *CI* confidence interval, *PGY* postgraduate year, *PC* patient care, *MK* medical knowledge, *SBP* systems-based practice, *PBLI* practice-based learning and improvement, *PROF* professionalism, *ICS* interpersonal and communication skills

^*^P < 0.05

^a^Described as mean (standard deviation)

## Discussion

This cross-sectional study found that residents generally rated themselves significantly higher than CCC faculty on Anesthesiology Milestones. Medical education degree and domain of competency were independently associated with the differences between resident self- and faculty- assessments.

This study provided emerging evidence that anesthesia residents tended to overestimate their own competence, which might affect their clinical judgements. Anesthesiologists often face unexpected crisis events during clinical practice. A key element of crisis management is decidedly calling for help when needed. Therefore, it is of utmost importance to cultivate the ability of self-assessment during residency training, which can help residents know whether the current clinical situation is beyond their capacity and when additional help is required. To our knowledge, there has been only limited data regarding the accuracy of anesthesia residents’ self-assessments. Ross FJ et al. demonstrated a strong agreement between resident self- and faculty-assessments on Anesthesiology Milestones 1.0 [16]. However, Fleming M et al. found that anesthesia residents’ ratings were lower than the faculty’s on a 5-point anchored Likert scale that was designed to evaluate overall clinical performance [7]. Previous studies have also shown conflicting results regarding the consistency between self- and faculty-assessments on ACGME Milestones among residents in other specialities, including surgery, ophthalmology, emergency medicine, and family medicine [8, 9, 12–15, 17].

There were some potential limitations in Milestones evaluation and feedback that may cause inaccurate assessments in our program. Faculty assessed residents using different methods across programs. In our program, CCC faculty discussed ratings based on their own impressions, daily assessments from supervisors, and objective examination scores. In some other programs, CCC faculty reviewed available 360° evaluations on residents’ performance [9, 15, 16]. The latter method should be more objective, since CCC faculty possibly lacked opportunities to observe residents’ behaviors in all the subcompetencies and thus needed supplemental evidence, such as recent evaluations from other physicians, nurses, peers, or patients. Furthermore, residents have regularly received Milestone-based feedback for more than five years in the US [23, 24], while the Milestone Evaluation System has just been promoted in China. Therefore, our residents cannot understand the descriptions of Milestones as adequately as the US residents, which might contribute to their overestimation. This can be improved using the “ask-tell-ask” feedback method, in which faculty ask residents to perform self-assessments first, inform them of faculty-assessments next, and finally discuss action plans together [25, 26].

This study suggested the differences between self- and faculty-assessments varied across competencies. Residents in our program overestimated their own competency in PC, PBLI, and PROF (Fig. 1). The self- and faculty-assessments were less different in MK and ICS, when compared with PC (Table 3), aligning with findings of a study in ophthalmology residents [9]. A plausible explanation is that our residents received feedback of their written examination scores every three months in our program, providing them with a comprehensive understanding of their progress in MK competency. Similarly, residents could assess their own ICS competency by considering others’ attitudes towards them and feedback received during their interactions. Conversely, competencies of PBLI and PROF were not frequently remarked upon by supervisors or discussed among peers. Some residents acknowledged the significance of PBLI and committed considerable time to this endeavor. Nevertheless, their insufficient self-learning skills impeded them from achieving the desired learning outcomes. This kind of residents was prone to overestimating their proficiency in PBLI, possibly due to a confusion between their efforts and actual competency.

Our study revealed that residents with higher medical degrees tended to have ratings closer to the faculty's assessments compared to those with a bachelor's degree. On one hand, residents with advanced medical degrees received higher scores in National Entrance Examinations, indicating better academic performance that may be associated with enhanced self-awareness abilities [27]. On the other hand, their graduate education, which includes clinical rotations and research training, could effectively strengthen their abilities for self-awareness.

Accumulating evidence has illustrated that female residents were more likely to underestimate themselves than male residents in surgery programs [14, 15, 28], which was not supported by our data. It is worth noting that females accounted for approximately 70% of the residents, 80% of the CCC members, and 60% of all the faculty in our department; hence, female anesthesiologists were not at a disadvantage, which was a major difference from surgeons. We also did not observe a significant association between the differences and grade of resident or ranks of written examination scores. Some studies found a tendency of underestimation in senior residents and overestimation in junior residents [29, 30]. This was explained by the metacognitive bias known as the Dunning-Kruger Effect, which means the least skilled individuals tend to be the most overconfident [3]. However, the Dunning-Kruger Effect has been questioned recently, as poor performers lack cognitive resources to assess their ability and may therefore either overestimate or underestimate themselves [31].

This study had the following limitations. First, the cross-sectional design limited our ability to draw a causal conclusion; thus, future studies are required to validate the significant associations found in our study. Second, the accuracy of self- and faculty-assessments may be influenced by factors such as the experience and number of CCC faculty, the methodology of evaluation, and the feedback provided to residents. In addition, variations in medical education programs and residency training across countries may restrict the generalizability of our findings to other centers, countries, or specialties. Finally, this study did not investigate deep reasons behind the differences between resident- and faculty-assessments, since some potentially associated factors could not be summarized as quantitative data. Interviews with residents and faculty can provide more detailed information.

## Conclusions

Our study revealed differences between resident self- and faculty-assessments on Anesthesiology Milestones among anesthesia residents in China. The differences between them were associated with residents’ medical education degrees and domains of competency. These findings emphasized the need to improve the accuracy of Milestones self-assessment, especially in residents with bachelor’s degree and in competencies of PC, PBLI and PROF.

## Data Availability

The datasets used and/or analyzed during the current study are available from the corresponding author on reasonable request.

## Abbreviations

ACGME: Accreditation Council for Graduate Medical Education
US: United States
PC: Patient care
MK: Medical knowledge
SBP: System-based practice
PBLI: Practice-based learning and improvement
PROF: Professionalism
ICS: Interpersonal and communication skills
CCC: Clinical Competency Committee
PUMCH: Peking Union Medical College Hospital
ICC: Intraclass correlation coefficient
GEE: Generalized estimating equation
SD: Standardized deviation
CI: Confidence interval
